# Integrating Ergonomic Risk Assessment with the Hierarchy of Controls Among Informal Sewing Workers in Rural Thailand

**DOI:** 10.3390/healthcare14070828

**Published:** 2026-03-24

**Authors:** Ratchanee Joomjee, Monthicha Raksilp, Niruwan Turnbull, Ruchakron Kongmant, Watthanasak Jeamwatthanachai, Wipa Chuppawa

**Affiliations:** 1 Faculty of Public Health, Ubon Ratchathani Rajabhat University, Ubon Ratchathani 34190, Thailand; ratchanee.j@ubru.ac.th (R.J.); monthicha.r@ubru.ac.th (M.R.); 2 Public Health and Environmental Policy in Southeast Asia Research Cluster (PHEP-SEA), Mahasarakham University, Maha Sarakham 44150, Thailand; phepsea.msu@gmail.com; 3 Faculty of Public Health, Mahasarakham University, Maha Sarakham 44150, Thailand; 4 Cognitive Computing and Human-Machine Collaboration Core Artificial Intelligence, National Electronics and Computer Technology Center (NECTEC), Pathum Thani 12120, Thailand; watthanasak.jea@nectec.or.th; 5 Faculty of Liberal Arts and Science, Sisaket Rajabhat University, Mueang District, Sisaket 33000, Thailand; wipa.c@sskru.ac.th

**Keywords:** informal workers, sewing workers, ergonomics, work-related musculoskeletal disorders, mental workload, NASA-TLX, Rapid Upper Limb Assessment, hierarchy of ergonomic controls, occupational health

## Abstract

**Highlights:**

**What are the main findings?**

- Informal sewing workers exhibited high ergonomic risk (mean RULA = 6.91; 87.3% at Action Level 4) and high WMSD prevalence, particularly in the neck, shoulders, and upper back.
- Significant correlations were observed between ergonomic risk and both mental and physical workload, indicating a combined biomechanical and psychosocial burden.

**What are the implications of the main findings?**

- Engineering and administrative controls within the Hierarchy of Controls are the most effective and feasible strategies to reduce ergonomic risks in informal settings.
- Integrated, participatory ergonomic interventions are essential to sustainably prevent WMSDs and improve occupational health among informal workers in low-resource contexts.

**Abstract:**

**Background:** Informal sewing workers are widely exposed to ergonomic and workload-related risks but remain largely excluded from formal occupational health protection, particularly in low- and middle-income countries. This study evaluated integrated physical and mental workload risks associated with WMSDs among informal sewing workers to develop contextually feasible preventive guidelines based on the Hierarchy of Ergonomic Control. **Methods:** A mixed-methods study was conducted among 150 informal sewing workers in Ubon Ratchathani Province, Thailand. Quantitative data were collected using a structured questionnaire, the Rapid Upper Limb Assessment (RULA), the Nordic Musculoskeletal Questionnaire (NMQ), and the NASA Task Load Index (NASA-TLX). Associations between sociodemographic characteristics, ergonomic risks, and WMSDs were analyzed using chi-square tests and correlation analysis. Qualitative data were obtained through a focus group discussion with key stakeholders to develop ergonomic control strategies guided by the HEC framework. **Results:** The majority of participants were female and middle-aged, with widespread exposure to high-risk ergonomic conditions, including prolonged sitting, repetitive tasks, and awkward postures. A high prevalence of WMSDs was observed, particularly in the neck, shoulders, and back. Younger workers and those with lower educational attainment experienced significantly higher ergonomic risk exposure and WMSD prevalence. NASA-TLX results indicated that physical demand and performance pressure were the main contributors to overall workload. Application of the HEC framework showed that elimination and substitution controls were the most effective strategies for reducing ergonomic risks, followed by engineering controls, while administrative measures and personal protective equipment were less effective. **Conclusions:** Informal sewing workers face substantial ergonomic and mental workload risks that contribute to a high burden of WMSDs. Prioritizing higher-order ergonomic controls, integrating workload management, and implementing community-based ergonomic interventions are essential to improving occupational health and reducing inequities among informal workers.

## 1. Introduction

Informal employment represents a substantial proportion of the global workforce, accounting for approximately two billion workers worldwide, with nearly 88% located in Asia [1,2]. In low- and middle-income countries (LMICs), informal workers frequently operate outside formal regulatory frameworks and lack access to occupational health protection. As a result, they are disproportionately exposed to hazardous working conditions, particularly ergonomic risks. Among these groups, informal sewing workers constitute a highly vulnerable population due to prolonged static postures, repetitive upper-limb movements, poor workstation design, and production-driven workload pressures [3,4,5,6,7].

Musculoskeletal disorders (MSDs) are consistently reported as the most prevalent occupational health outcome among sewing machine operators. Studies from India, Indonesia, Nigeria, and Sri Lanka have identified high rates of neck, shoulder, and lower back pain, largely attributable to sustained awkward postures and repetitive tasks [4,5,6,7,8]. Objective ergonomic assessment tools such as the Rapid Upper Limb Assessment (RULA), Rapid Entire Body Assessment (REBA), and Ovako Working Posture Analysis System (OWAS) have been widely used to quantify biomechanical exposure [9,10]. These tools effectively identify high-risk postures and provide action-level classifications; however, they primarily assess physical loading and do not capture the cognitive and performance-related demands inherent in garment production.

Workload in sewing operations is multidimensional, comprising both physical and mental components. In informal rural contexts, workers must simultaneously maintain speed, precision, and quality while managing economic uncertainty and production quotas. The NASA Task Load Index (NASA-TLX) has been applied across industrial and healthcare sectors to evaluate perceived workload across six dimensions: mental demand, physical demand, temporal demand, performance, effort, and frustration [11,12,13]. Emerging evidence suggests that mental workload may exacerbate biomechanical strain through sustained attention, time pressure, and performance expectations, thereby increasing vulnerability to work-related musculoskeletal disorders (WMSDs) [14,15]. Despite this evidence, most ergonomic studies in the informal garment sector examine physical and mental risks independently, limiting the ability to understand their combined or synergistic effects.

Beyond risk identification, a more critical gap concerns intervention prioritization. In resource-limited informal settings, the central occupational health challenge is not only determining whether risks exist, but deciding which control measures should be implemented first. The Hierarchy of Controls comprising elimination, substitution, engineering controls, administrative controls, and personal protective equipment (PPE) is widely recognized as a structured framework for ranking intervention effectiveness [16,17]. While this model is extensively applied in formal industrial environments, its operationalization in informal rural labor settings remains underdeveloped. Moreover, ergonomic assessments rarely translate quantified risk scores into graded, feasibility-informed control strategies.

Consequently, an important decision-making problem persists: how can objective postural risk, subjective workload perception, and structured intervention prioritization be systematically integrated to guide practical and contextually feasible ergonomic improvements among informal workers? To address this gap, the present study advances a structured integration framework linking: (1) objective biomechanical exposure (RULA), (2) subjective multidimensional workload assessment (NASA-TLX), and (3) prioritized intervention strategies using the Hierarchy of Ergonomic Controls (HEC). Rather than employing these tools in isolation, this study synthesizes musculoskeletal symptom prevalence (NMQ), postural strain (RULA), and cognitive workload (NASA-TLX) to create an evidence-based pathway from risk identification to control prioritization. This integration enables a transition from descriptive risk reporting to actionable decision support, particularly in economically constrained informal rural environments.

Focusing on informal sewing workers in Ubon Ratchathani Province, Thailand, this research contributes in three distinct ways. First, it provides an integrated assessment of physical and mental workload in a predominantly female, rural informal workforce largely excluded from occupational health governance. Second, it demonstrates how multidimensional ergonomic risk data can be mapped onto a graded control hierarchy to support intervention prioritization. Third, it offers contextually feasible, low-cost ergonomic recommendations grounded in stakeholder consensus and aligned with socio-economic realities. By bridging ergonomic risk quantification with structured control prioritization, this study extends occupational health research beyond isolated exposure measurement and toward an integrated decision-support framework tailored to informal labor settings in LMICs.

## 2. Materials and Methods

### 2.1. Study Design and Setting

This study employed a mixed-methods design conducted between October and December 2022 in Muang Sam Sip District, Ubon Ratchathani Province, Thailand. The study area was selected because it contains a high concentration of home-based and community-based informal sewing workers and because collaboration with local public health authorities enabled detailed ergonomic field assessments. The research consisted of two sequential components: (1) a quantitative cross-sectional assessment of ergonomic risk exposure, mental workload, and work-related musculoskeletal disorders (WMSDs); and (2) a qualitative focus group discussion (FGD) to develop contextually feasible ergonomic control strategies guided by the Hierarchy of Ergonomic Controls (HEC). The study was designed as a district-based occupational health investigation rather than a multi-province or nationally representative survey.

### 2.2. Sampling Frame and Participant Recruitment

#### 2.2.1. Sampling Frame

The sampling frame comprised informal sewing workers operating within three sub-districts of Muang Sam Sip District: Yang Sak Krapho Lum, Phai Yai, and Yang Yo Phap. Because informal workers are not registered within a formal employment database, eligible participants were identified through: (1) sub-district administrative organizations, (2) community leaders, (3) local health promotion officers, and (4) sewing group representatives. These sources provided community-level listings of active informal sewing workers.

#### 2.2.2. Eligibility Criteria

Inclusion criteria:

(1) Aged 18 years or older;
(2) Engaged in informal sewing work for at least 6 months;
(3) Working primarily in home-based or community-based informal settings performing sewing-related tasks for ≥ 4 h per day.

Exclusion criteria:

(1) Formal factory employees;
(2) Individuals with diagnosed traumatic musculoskeletal injuries unrelated to sewing work;
(3) Individuals unable to complete questionnaires or workload assessment procedures.

These criteria ensured adequate exposure duration and homogeneity of informal occupational conditions.

#### 2.2.3. Sample Size Determination

The required sample size was calculated using the single population proportion formula based on a previously reported WMSD prevalence of 70%, a 95% confidence level (Z = 1.96), and a margin of error of 7.5% [18]. The calculated minimum sample was 144 participants. To account for potential non-response and incomplete data, the final sample size was set at 150 participants.

#### 2.2.4. Quota Sampling and Rationale

Quota sampling was applied to ensure balanced representation across the three sub-districts (50 participants per sub-district). This approach was selected for the following reasons: (1) to prevent over-representation from a single easily accessible sewing cluster; (2) to capture variability between dispersed home-based workers and organized community sewing groups; to ensure geographic distribution within the district; and to maintain feasibility for direct ergonomic observation and video-based RULA assessment. Quota allocation was intended to enhance internal variability rather than achieve statistical representativeness at provincial or national levels.

#### 2.2.5. Recruitment Pathway

Eligible workers were approached through community leaders and local health officers. Researchers visited workplaces to explain the study objectives and procedures. Participation was voluntary, and written informed consent was obtained prior to data collection.

### 2.3. Study Instruments and Data Collection

Data collection was conducted on-site at participants’ workplaces during regular working hours to ensure ecological validity.

#### 2.3.1. Sociodemographic and Occupational Questionnaire

A structured questionnaire was developed to collect data on demographic characteristics (age, gender, education, and income); occupational history and working conditions; and exposure to ergonomic risk factors (e.g., prolonged sitting, bending, repetitive tasks, and workload pressure). Content validity was reviewed by three occupational health experts. A pilot test was conducted with 20 informal sewing workers outside the study area to ensure clarity and feasibility. Necessary revisions were made before full implementation.

#### 2.3.2. Nordic Musculoskeletal Questionnaire (NMQ)

WMSDs were assessed using the standardized Thai version of the Nordic Musculoskeletal Questionnaire (NMQ). Participants reported pain, discomfort, or numbness in nine anatomical regions during the previous 12 months. A case of WMSD was defined as symptoms lasting at least 24 h that interfered with work activities [12].

#### 2.3.3. Rapid Upper Limb Assessment (RULA)

**Figure 1:**
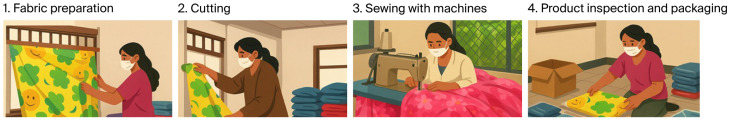
Analysis of the sewing process.

Postural risk was evaluated using RULA. Four core sewing-related tasks were assessed: fabric preparation; cutting; machine sewing (approximately 4 h/day); and product inspection and packaging. Each participant was video-recorded for at least 10 min during a complete sewing cycle. The posture demonstrating the greatest joint deviation or longest static load was selected for scoring. Two trained researchers independently performed RULA scoring. Inter-rater reliability was assessed using Cohen’s kappa (κ = 0.82), indicating strong agreement. RULA scores (1–7) were categorized into four action levels requiring varying degrees of corrective action [19,20]. Four core sewing-related tasks were analyzed: (1) fabric preparation, (2) cutting, (3) machine sewing (approximately 4 h/day), and 4) product inspection and packaging (Figure 1 and Table 1). Each participant was video-recorded for at least 10 min during a full sewing cycle. The worst posture, defined as the posture with the most extreme joint deviation or the longest duration of static load, was selected for analysis from the video frames. Data were analyzed using the RULA scoring system to determine the action level required [21].

#### 2.3.4. NASA Task Load Index (NASA-TLX)

Mental workload was assessed using the Thai-validated version of the NASA-TLX, which measures six dimensions: Mental demand, Physical demand, Temporal demand, Performance, Effort, and Frustration. The standard three-step procedure was applied: (1) pairwise comparison to determine weighting of dimensions; (2) rating each dimension on a 0–20 scale; and Calculation of Weighted Workload (WWL) and average TLX score. Workload scores were categorized into five levels: very low, low, medium, high, and very high. NASA-TLX assessments were administered immediately after task performance to minimize recall bias [22].

### 2.4. Qualitative Component

Following quantitative data collection, one focus group discussion (FGD) was conducted with 16 purposively selected stakeholders: (1) community leaders (*n* = 6), (2) sewing group representatives (*n* = 6), and (3) health promotion officers (*n* = 4). The FGD aimed to translate quantitative findings into feasible ergonomic interventions aligned with the Hierarchy of Ergonomic Controls (HEC). The session lasted approximately two hours, was audio-recorded with consent, and transcribed verbatim [22].

### 2.5. Data Analysis

Quantitative data were analyzed using SPSS version 19. Descriptive statistics summarized demographic characteristics, ergonomic exposures, workload levels, and WMSD prevalence. Chi-square and Fisher’s exact tests examined associations between socio- demographic factors and ergonomic risks or WMSDs. Pearson’s correlation assessed relationships between RULA scores and NASA-TLX dimensions. Statistical significance was set at *p* < 0.05. Qualitative data were analyzed using thematic analysis. Two researchers independently coded transcripts. Emergent themes were mapped to the five levels of the Hierarchy of Ergonomic Controls. Discrepancies were resolved through consensus discussion.

### 2.6. Ethical Considerations

The study was approved by the Ubon Ratchathani Rajabhat University Ethics Committee (Reference No. HE652033-031/2565). All participants provided written informed consent. Participation was voluntary, and confidentiality was strictly maintained through data anonymization.

## 3. Results

### 3.1. Participant Characteristics and Ergonomic Risk Factors

Table 2 presents the sociodemographic characteristics and work-related risk profiles of informal workers included in the study (*n* = 150). The sample was predominantly female, with 141 participants (94.0%), while males accounted for only 6.0% (*n* = 9). Regarding age distribution, nearly half of the participants were aged 50–60 years (48.0%, *n* = 72), followed by those aged < 50 years (34.7%, *n* = 52) and > 60 years (17.3%, *n* = 26). The mean age of the participants was 53.41 years (SD = 7.78), with an age range spanning from 31 to 76 years, indicating a predominantly middle-aged to older workforce. In terms of educational attainment, the majority of participants had completed more than high school education (62.0%, *n* = 93), while 38.0% (*n* = 57) had an education level below high school. With respect to income, most participants reported earning more than 5000 baht per month (85.3%, *n* = 128), whereas 14.7% (*n* = 22) reported a monthly income below this threshold. The assessment of high-risk work characteristics revealed widespread exposure to ergonomic risk factors. Prolonged sitting for more than two hours was reported by 86.7% of participants (*n* = 130). Almost all workers reported head bending or lifting (98.0%, *n* = 147), twisting or tilting the body (93.3%, *n* = 140), and repetitive tasks (93.3%, *n* = 140). Additionally, forward bending was reported by 91.3% (*n* = 137), and lifting heavy objects by 71.3% (*n* = 107). A substantial proportion of participants indicated working hurriedly (80.7%, *n* = 121), while excessive workload was reported by 28.7% (*n* = 43). Notably, nearly all participants experienced uncertain income (98.0%, *n* = 147), highlighting economic instability within this informal workforce (Table 2).

Table 3 demonstrates that age group was significantly associated with sitting for more than 2 h (χ^2^ = 6.51, *p* = 0.025) and perception of sewing as a cause of pain (χ^2^ = 35.019, *p* < 0.001), although odds ratios were not calculated due to zero cell frequencies (95% CI: 0.734–0.872 and 0.087–0.202, respectively). No significant associations were observed between age and other ergonomic risk factors, including head bending/lifting, forward bending, twisting/tilting, repetitive tasks, lifting heavy objects, working hurriedly, complex tasks, excessive workload, or uncertain income (*p* > 0.05). In contrast, education level showed broader associations with ergonomic exposures, including sitting for more than 2 h (χ^2^ = 14.14, *p* < 0.001; 95% CI: 0.482–0.654), forward bending (χ^2^ = 8.724, *p* = 0.002; 95% CI: 0.507–0.673), twisting/tilting (χ^2^ = 6.567, *p* = 0.014; 95% CI: 0.517–0.680), repetitive tasks (χ^2^ = 4.657, *p* = 0.043; OR = 0.238, 95% CI: 0.059–0.962), complex tasks (χ^2^ = 7.275, *p* = 0.007; 95% CI: 1.476–1.947), and perception of sewing as a cause of pain (χ^2^ = 11.980, *p* = 0.001; 95% CI: 0.280–0.437). Borderline associations were observed for head bending/lifting (*p* = 0.053) and lifting heavy objects (*p* = 0.063; OR = 2.194, 95% CI: 1.001–4.808), while no significant associations were identified for working hurriedly, excessive workload, or uncertain income (*p* > 0.05) (Table 3).

### 3.2. Prevalence of Work-Related Musculoskeletal Disorders (WMSDs)

Table 4 shows that age group was significantly associated with musculoskeletal pain in the neck (χ^2^ = 14.411, *p* = 0.001; OR = 5.123, 95% CI: 2.095–12.528), shoulder (χ^2^ = 8.756, *p* = 0.006; OR = 3.593, 95% CI: 1.491–8.654), and upper back (χ^2^ = 8.955, *p* = 0.005; OR = 3.610, 95% CI: 1.509–8.636), indicating higher odds of pain among older participants. No significant associations were observed between age and lower back, upper arm, lower arm, elbow, hand/wrist, hip, knee, calf, or foot pain (*p* > 0.05). In contrast, education level demonstrated significant associations with neck pain (χ^2^ = 9.600, *p* = 0.003; OR = 0.306, 95% CI: 0.142–0.659), shoulder pain (χ^2^ = 4.868, *p* = 0.035; OR = 0.440, 95% CI: 0.211–0.920), upper back pain (χ^2^ = 9.034, *p* = 0.004; OR ≈ 0.344, 95% CI: 0.170–0.698), lower back pain (χ^2^ = 5.780, *p* = 0.024; OR = 0.438, 95% CI: 0.222–0.863), hand/wrist pain (χ^2^ = 13.752, *p* < 0.001; OR = 0.250, 95% CI: 0.117–0.532), hip pain (χ^2^ = 19.915, *p* < 0.001; OR = 0.205, 95% CI: 0.100–0.420), and knee pain (χ^2^ = 7.208, *p* = 0.009; OR = 0.395, 95% CI: 0.199–0.784), suggesting lower odds of WMSDs among participants with higher education. No statistically significant associations were identified between education level and lower arm, elbow, calf, or foot pain (*p* > 0.05) (Table 4).

### 3.3. Mental Workload Assessment (NASA-TLX)

Table 5 presents the summary of perceived workload as assessed using the NASA Task Load Index (NASA-TLX) among informal sewing workers (*n* = 150). The analysis includes weighted workload levels (WWL), risk categorization, mean scores with standard deviations, and correlation coefficients. Overall, the performance dimension demonstrated the highest workload burden, with a WWL score of 77.90, corresponding to a high risk level. This indicates that workers perceived substantial demands related to task performance requirements. Despite its high workload classification, the performance subscale did not show a statistically significant correlation (r = 0.081, *p* = 0.325). The physical demand dimension exhibited a medium risk level, with a WWL of 51.43 and a mean score of 46.44 ± 27.36. Physical demand was significantly correlated with overall workload outcomes (r = 0.312, *p* < 0.001), suggesting that physical strain constitutes a key contributor to perceived workload among informal sewing workers. In contrast, mental demand, temporal demand, and effort were classified as low risk levels, with WWL values of 37.97, 32.07, and 36.53, respectively. Among these, mental demand demonstrated a statistically significant but weak positive correlation (r = 0.171, *p* = 0.037), while temporal demand (r = −0.132, *p* = 0.108) and effort (r = −0.151, *p* = 0.065) did not reach statistical significance. The frustration subscale recorded the lowest workload contribution, with a WWL of 4.63 and a very low risk level. The mean frustration score was 4.90 ± 10.00, and no significant correlation was observed (r = 0.024, *p* = 0.768) (Table 5).

### 3.4. Hierarchy of Ergonomic Controls (HEC)

Table 6 summarizes the implementation of the Hierarchy of Ergonomic Controls (HEC) applied to prevent and mitigate work-related musculoskeletal disorders (WMSDs) among informal sewing workers. The findings illustrate a graded approach to ergonomic risk reduction, with varying levels of effectiveness across control strategies. At the highest level, elimination controls were identified as the most effective intervention. High-risk postures associated with WMSDs—such as forward bending, prolonged sitting, awkward working positions, and manual handling of heavy objects—were substantially reduced through the establishment and enforcement of workplace safety standards. These measures demonstrated a high level of effectiveness, indicating that direct removal of ergonomic hazards plays a critical role in reducing musculoskeletal risk. Substitution controls showed a moderately high effectiveness. The replacement of non-ergonomic seating and equipment with physiologically supportive alternatives, including cushioned seat pads and chairs with appropriate lumbar and arm support, contributed to improved physical comfort and a reduced likelihood of musculoskeletal injury. At the engineering control level, ergonomic workstation redesign based on workers’ anthropometric dimensions was implemented. This included the use of ergonomically appropriate tables and chairs, optimization of sewing area layouts to facilitate efficient movement, and the introduction of preventive maintenance schedules to ensure reliable machine performance. These engineering interventions demonstrated a moderate level of effectiveness in reducing unnecessary physical exertion during work activities. Administrative controls were associated with a moderately low level of effectiveness. Interventions included restructuring work schedules to limit continuous sitting to no more than two hours, introducing regular rest breaks tailored to individual physical capacity, implementing targeted stretching exercises for frequently affected muscle groups (e.g., neck, shoulders, back, knees, and feet), and applying the 5S methodology to improve workspace organization. Clear safety protocols were also established to promote safe work practices. Finally, personal protective equipment (PPE) represented the lowest tier of control and demonstrated low effectiveness. PPE was primarily used to reduce localized vibration exposure, particularly to the feet during sewing tasks, and to provide additional ergonomic support. While beneficial as a supplementary measure, PPE alone offered limited protection compared with higher-level control strategies (Table 6).

### 3.5. Integration of Ergonomic Risk Assessment, Workload, and Control Strategies

**Figure 2:**
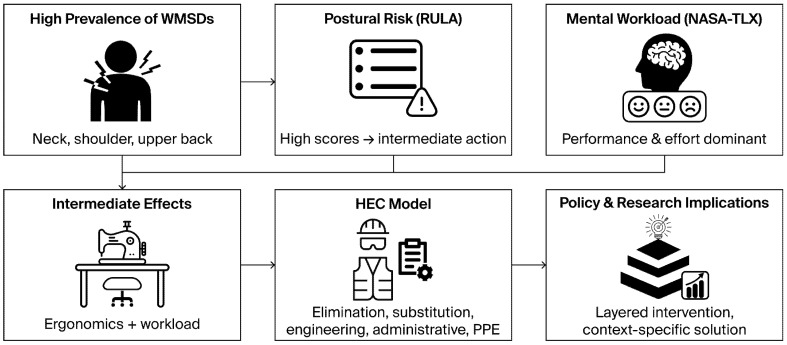
Linking ergonomic risks, workload, and the HEC model.

Figure 2 illustrates the relationship between ergonomic risk exposure, workload characteristics, and control strategies among informal sewing workers. Figure 2 summarizes the ergonomic risk assessment, showing widespread exposure to prolonged sitting, forward bending, repetitive movements, and awkward postures involving the neck, trunk, and upper limbs, indicating elevated biomechanical risk across routine sewing tasks. The figure also integrates these identified ergonomic hazards with mental workload dimensions, particularly physical and performance demands, and maps them onto the Hierarchy of Ergonomic Controls (HEC) model, demonstrating how higher-order controls address primary ergonomic risks while lower-level controls manage residual exposure. Together, the figures depict a structured progression from ergonomic risk identification to risk management within the study framework (Figure 2).

## 4. Discussion

This study examined associations between ergonomic risk exposure, perceived workload, and work-related musculoskeletal disorders (WMSDs) among informal sewing workers in rural Thailand. By integrating postural risk assessment (RULA), self-reported musculoskeletal symptoms (NMQ), mental workload evaluation (NASA-TLX), and a stakeholder-informed application of the Hierarchy of Ergonomic Controls (HEC), the study provides an associative and context-specific understanding of occupational risk patterns in an informal labour setting. Given the cross-sectional design, the findings should be interpreted as evidence of relationships rather than causal effects.

### 4.1. Ergonomic Risk Exposure in Informal Sewing Work

The high prevalence of prolonged sitting, repetitive movements, forward bending, and sustained neck and trunk deviation observed in this study is consistent with previous research in textile and garment sectors [5,6,7,8]. These exposure patterns reflect the biomechanical characteristics of sewing work, particularly in home-based environments where workstation design is often not ergonomically optimized. Although the co-occurrence of high-risk postures and reported WMSDs aligns with established ergonomic literature [9], the present design does not permit causal attribution. Instead, the findings indicate that musculoskeletal symptoms and ergonomic exposures are statistically associated within this workforce. These patterns highlight structural occupational vulnerabilities rather than demonstrating direct injury mechanisms. The predominance of female participants reflects gendered labour segmentation within informal economies [1,2]. This demographic profile may influence exposure characteristics and access to ergonomic modifications; however, gender-specific inferences remain limited due to the small number of male participants.

### 4.2. Sociodemographic Associations with WMSDs

Educational attainment showed significant associations with both ergonomic exposures and WMSD prevalence across multiple anatomical regions. Workers with lower educational levels were more likely to report high-risk work characteristics and musculoskeletal symptoms. These findings are consistent with literature suggesting that education may influence ergonomic literacy, risk awareness, and adaptive behaviour [3,9]. However, causality cannot be inferred; education likely interacts with broader socioeconomic determinants. The finding that younger workers (<60 years) reported higher prevalence of certain spinal WMSDs contrasts with assumptions that musculoskeletal disorders are primarily age-driven. This pattern may reflect differences in task intensity, workload distribution, or exposure duration within this informal setting. Without longitudinal exposure assessment, these associations should be interpreted cautiously. Overall, the results suggest that ergonomic risk and musculoskeletal burden are socially patterned within this workforce rather than solely biologically determined.

### 4.3. Mental Workload and Its Relationship with Ergonomic Risk

NASA-TLX results indicated that performance demands and physical demand contributed most prominently to perceived workload. Physical demand demonstrated a statistically significant correlation with overall workload scores, whereas other dimensions showed weaker or non-significant associations. These findings suggest that physical and performance-related demands coexist within informal sewing tasks. However, the magnitude of correlations was modest, and the cross-sectional design does not establish whether workload contributes to musculoskeletal strain or reflects adaptation to physical discomfort. Rather than indicating a causal pathway, the findings highlight the multidimensional nature of occupational burden in informal settings. Integrating mental workload assessment into ergonomic evaluation broadens the scope of risk characterization, particularly in tasks requiring sustained attention and production pressure.

### 4.4. Interpretation of the Hierarchy of Ergonomic Controls (HEC)

The graded control hierarchy presented in this study reflects theoretical prioritization based on established occupational safety principles [16,17] combined with stakeholder-derived feasibility judgments from the focus group discussion. It is important to clarify that the study did not experimentally test intervention outcomes. Elimination and substitution strategies were conceptually ranked higher because they target hazard sources directly. Engineering controls were considered feasible but resource-dependent. Administrative measures and personal protective equipment (PPE) were identified as supplementary strategies. These rankings reflect alignment with established safety frameworks and stakeholder perspectives, not measured effectiveness within this study. Accordingly, references to “effectiveness” should be interpreted as theoretical hierarchy alignment rather than demonstrated intervention impact.

### 4.5. Practical and Policy Considerations

Despite design limitations, the findings suggest several practical considerations: (1) ergonomic interventions in informal settings should prioritize hazard modification where economically feasible, (2) educational support may enhance ergonomic awareness among lower-educated workers, (3) workload management strategies should consider both physical and performance-related demands, and (3) community-based participatory approaches may enhance contextual relevance and adoption. These recommendations are derived from associative findings and stakeholder dialogue rather than intervention trials. Policy development should therefore consider them as preliminary guidance requiring further empirical validation.

### 4.6. Strengths, Limitations, and Future Research

A strength of this study is the structured integration of validated ergonomic and workload assessment tools within a conceptual control hierarchy. The mixed-methods approach enabled both quantitative characterization and qualitative contextualization. However, several limitations must be acknowledged: the cross-sectional design precludes causal inference, the single-district sample limits generalizability, the small male sample restricts gender comparison, and psychosocial stressors, comorbidities, and longitudinal exposure patterns were not comprehensively assessed. Future research should include longitudinal ergonomic intervention studies, cost-effectiveness analyses of workstation modifications, and evaluation of implemented HEC informed strategies. Objective posture-tracking technologies may further enhance measurement precision.

In summary, the study identifies substantial ergonomic exposures and associated musculoskeletal symptoms among informal sewing workers. The integration of RULA, NMQ, NASA-TLX, and the HEC framework provides a structured approach to understanding occupational risk and prioritizing theoretically aligned control strategies. However, findings reflect associations and stakeholder-informed feasibility rather than demonstrated intervention effectiveness. Further longitudinal and interventional research is required to determine the real-world impact of proposed control measures.

## 5. Conclusions

In summary, informal sewing workers experience substantial ergonomic and mental workload risks that contribute to a high burden of WMSDs. Addressing these challenges requires prioritizing higher-order ergonomic controls, integrating mental workload considerations, and embedding interventions within community-based and policy-supported frameworks. Such approaches are essential for reducing occupational health inequities among informal workers in low- and middle-income countries.

## Figures and Tables

**Table 1:** RULA score and level of WMSD risk.

Score	Level of WMSD Risk
1–2	Acceptable working posture if not maintained or repeated for long periods.
3–4	Further investigation is needed. Posture change may be required.
5–6	Investigate and implement posture changes soon to avoid further exposure to WMSD risk.
7	Requires immediate attention and changes in posture.

**Table 2:** The participant characteristics of informal workers (*n* = 150).

Characteristics Variables	Categories	*N*	Percentage
Gender	Male	9	6.0
	Female	141	94.0
Age group Mean = 53.41, SD = 7.780, Max = 76, Min = 31	<50 years	52	34.7
	50–60 years	72	48.0
	>60 years	26	17.3
Education	≤high school	57	38.0
	>high school	93	62.0
Income	<5000 baht per month (about $152)	22	14.7
	≥5000 baht per month	128	85.3
High-risk work	Sitting > 2 h	130	86.7
	Head bending/lifting	147	98.0
	Forward bending	137	91.3
	Twisting/tilting body	140	93.3
	Repetitive tasks	140	93.3
	Lifting heavy objects	107	71.3
	Working hurriedly	121	80.7
	Complex tasks	11	7.3
	Excessive workload	43	28.7
	Uncertain income	147	98.0

**Table 3:** Socio-demographic characteristics and specific ergonomic risk factors (*n* = 150).

Working Characteristics	Age Group		Chi-Square	*p*-Value	OR (95%CI)		Education		Chi-Square	*p*-Value	OR (95%CI)	
	<60 Years	≥60 Years					>High School	≤High School				
	*n* (%)	*n* (%)			Lower	Upper	*n* (%)	*n* (%)			Lower	Upper
(1) Sitting > 2 h			6.510	0.025 *	n/c (0.734 to 0.872)				14.140	0.000 **	n/c (0.482 to 0.654)	
-Yes	104 (69.3)	26 (17.3)					57 (38.0)	73 (48.7)				
-No	20 (13.3)	0 (0)					0 (0)	20 (13.3)				
(2) Head bending/lifting			0.642	1.000	n/c (0.764 to 0.887)				4.995	0.053	n/c (0.297 to 0.454)	
-Yes	121 (80.7)	26 (17.3)					54 (36.0)	93 (62.2)				
-No	3 (2.0)	0 (0)					3 (2.0)	0 (0)				
(3) Forward bending			2.984	0.126	n/c (0.747 to 0.879)				8.724	0.002 *	n/c (0.507 to 0.673)	
-Yes	111 (74.0)	26 (17.3)					57 (38.0)	80 (53.3)				
-No	13 (8.7)	0 (0)					0 (0)	13 (8.7)				
(4) Twisting/ tilting body			2.247	0.211	n/c (0.752 to 0.881)				6.567	0.014 *	n/c (0.517 to 0.680)	
-Yes	114 (76.0)	26 (17.3)					57 (38.0)	83 (55.3)				
-No	10 (6.7)	0 (0)					0 (0)	10 (6.7)				
(5) Repetitive tasks			2.247	0.211	n/c (0.752 to 0.881)				4.657	0.043 *	0.238 (0.059 to 0.962)	
-Yes	114 (76.0)	26 (17.3)					50 (33.3)	90 (60.0)				
-No	10 (6.7)	0 (0)					7 (4.7)	3 (2.0)				
(6) Lifting heavy objects			2.862	0.100	2.108 (0.878 to 5.062)				3.946	0.063	2.194 (1.001 to 4.808)	
-Yes	92 (61.3)	15 (10.0)					46 (30.7)	61 (40.7)				
-No	32 (21.3)	11 (7.3)					11 (7.3)	32 (21.3)				
(7) Working hurriedly			1.225	0.413	0.492 (1.37 to 1.766)				1.611	0.21	0.591 (0.261 to 1.338)	
-Yes	98 (65.3)	23 (15.3)					43 (28.7)	78 (52.0)				
-No	26 (17.3)	3 (2.0)					14 (9.3)	15 (10.0)				
(8) Complex tasks			2.489	0.213	n/c (1.136 to 1.332)				7.275	0.007 **	n/c (1.476 to 1.947)	
-Yes	11 (7.3)	0 (0.0)					0 (0.0)	11 (7.3)				
-No	113 (75.3)	26 (17.3)					57 (38.0)	82 (54.7)				
(9) Perception of sewing as a cause of pain			35.019	0.000 **	n/c (0.087 to 0.202)				11.980	0.001 **	n/c (0.280 to 0.437)	
-Yes	124 (82.7)	19 (12.7)					50 (33.3)	93 (62.0)				
-No	0 (0)	7 (4.7)					7 (4.7)	0 (0)				
(10) Excessive workload			0.544	0.480	0.714 (0.290 to 1.754)				0.979	0.355	1.438 (0.699 to 2.954)	
-Yes	34 (22.7)	9 (6.0)					19 (12.7)	24 (16.0)				
-No	90 (60.0)	17 (11.3)					38 (25.3)	69 (46.0)				
(11) Uncertain income			0.642	1.000	n/c (0.764 to 0.887)				1.876	0.288	n/c (0.538 to 0.696)	
-Yes	121 (80.6)	26 (17.3)					57 (38.0)	90 (60.0)				
-No	3 (2.0)	0 (0.0)					0 (0.0)	3 (2.0)				

* Significant level at 0.05, ** Significant level at 0.01, *p*-value as Fisher’s Exact Test, n/c = not calculated.

**Table 4:** Socio-demographic characteristics and the prevalence of WMSDs (*n* = 150).

Part of the Body	Age Group		Chi-Square	*p*-Value	OR (95%CI)		Education		Chi-Square	*p*-Value	OR (95%CI)	
	<60 Years	≥60 Years					>High School	≤High School				
	*n* (%)	*n* (%)			Lower	Upper	*n* (%)	*n* (%)			Lower	Upper
(1) Neck			14.411	0.001 **	5.123 (2.095 to 12.528)				9.600	0.003 **	0.306 (0.142 to 0.659)	
-Pain	101 (67.3)	12 (8.0)					35 (23.3)	78 (52.0)				
-No pain	23 (15.3)	14 (9.3)					22 (14.7)	15 (10.0)				
(2) Shoulder			8.756	0.006 **	3.593 (1.491 to 8.654)				4.868	0.035 *	0.440 (0.211 to 0.920)	
-Pain	97 (64.7)	13 (8.7)					36 (24.0)	74 (49.3)				
-No pain	27 (18.0)	13 (8.7)					21 (14.0)	19 (12.7)				
(3) Upper back			8.955	0.005 **	3.610 (1.509 to 8.636)				9.034	0.004 **	3.44 (0.170 to 0.698)	
-Pain	90 (60.0)	11 (7.3)					30 (20.0)	71 (47.3)				
-No pain	34 (227.7)	15 (10.0)					27 (18.0)	22 (14.7)				
(4) Lower back			1.703	0.268	1.756 (0.749 to 4.113)				5.780	0.024 *	0.438 (0.222 to 0.863)	
-Pain	79 (52.7)	13 (8.7)					28 (18.7)	64 (42.7)				
-No pain	45 (30.0)	13 (8.7)					29 (19.3)	29 (19.3)				
(5) Upper arm			0.301	0.663	1.269 (0.542 to 2.969)				3.453	0.087	0.531 (0.271 to 1.039)	
-Pain	74 (49.3)	14 (9.3)					28 (18.7)	60 (40.0)				
-No pain	50 (33.3)	12 (8.0)					29 (19.3)	33 (22.0)				
(6) Lower arm			2.107	0.222	2.276 (0.732 to 7.073)				0.767	0.449	0.711 (0.331 to 1.527)	
-Pain	36 (24.2)	4 (2.7)					13 (8.7)	27 (18.1)				
-No pain	87 (58.4)	22 (14.8)					44 (29.5)	64 (43.6)				
(7) Elbow			0.113	0.790	0.840 (0.305 to 2.317)				1.554	0.303	0.583 (0.248 to 1.370)	
-Pain	26 (17.4)	6 (4.0)					9 (6.0)	23 (15.4)				
-No pain	98 (65.8)	19 (12.8)					47 (31.5)	70 (47.0)				
(8) Hand/Wrist			0.380	0.661	1.320 (0.906 to 3.193)				13.752	0.000 **	0.250 (0.117 to 0.532)	
-Pain	51 (34.0)	9 (6.0)					12 (8.0)	48 (32.0)				
-No pain	73 (48.7)	17 (11.3)					45 (30.0)	45 (30.0)				
(9) Hip			1.026	0.389	1.552 (0.660 to 3.646)				19.915	0.000 **	0.205 (0.100 to 0.420)	
-Pain	66 (44.0)	11 (7.3)					16 (10.7)	61 (40.7)				
-No pain	58 (38.7)	15 (10.0)					41 (27.3)	32 (21.3)				
(10) Knee			1.046	0.374	1.558 (0.663 to 3.662)				7.208	0.009	0.395 (0.199 to 0.784)	
-Pain	80 (53.3)	14 (9.3)					28 (18.7)	66 (44.0)				
-No pain	44 (29.3)	12 (8.0)					29 (19.3)	27 (18.0)				
(11) Calf			0.026	1.000	0.929 (0.383 to 2.256)				0.755	0.388	0.739 (0.374 to 1.463)	
-Pain	79 (52.7)	17 (11.3)					34 (22.7)	62 (41.3)				
-No pain	45 (30.0)	9 (6.0)					23 (15.3)	31 (20.7)				
(12) Foot			1.046	0.374	1.558 (0.663 to 3.662)				0.895	0.387	0.721 (0.366 to 1.421)	
-Pain	80 (53.3)	14 (9.3)					33 (22.0)	61 (40.7)				
-No pain	44 (29.3)	12 (8.0)					24 (16.0)	32 (21.3)				

* Significant level at 0.05, ** Significant level at 0.01, *p*-value as Fisher’s Exact Test.

**Table 5:** NASA TLX summary for informal sewing workers (*n* = 150).

Subscale	Weight	Rating	WWL	Risk Level	Mean ± SD	Coefficients (r)	*p*-Value
Mental	2.60	14.37	37.97	Low	32.18 ± 21.21	0.171	0.037 *
Physical	3.47	15.90	51.43	Medium	46.44 ± 27.36	0.312	<0.001 **
Temporal	2.43	13.50	32.07	Low	31.21 ± 16.35	−0.132	0.108
Performance	4.23	18.37	77.90	High	73.80 ± 21.78	0.081	0.325
Effort	2.27	16.67	36.53	Low	41.44 ± 21.55	−0.151	0.065
Frustration	0.40	7.63	4.63	Very Low	4.90 ± 10.00	0.024	0.768

* Significant level at 0.05, ** Significant level at 0.01.

**Table 6:** Hierarchy of ergonomics controls for WMSDs.

Control	HEC Controls for WMSDs	Effectiveness
Elimination	High-risk postures associated with musculoskeletal disorders (MSDs), such as bending, prolonged sitting, awkward positions, and manual handling of heavy objects. The establishment of workplace safety standards helped reduce these risks significantly.	high
Substitution	Selecting chairs and equipment that support physiology, such as cushioned seat pads and chair arms that provide lumbar support, decreases the risk of musculoskeletal injury and enhances physical comfort.	moderately high
Engineering	Designing the workstations for anthropometric dimensions, incorporating ergonomically appropriate tables and chairs. The spatial layout of sewing areas was optimized for efficiency and ease of movement. Preventive maintenance schedules were also implemented to ensure machinery functioned reliably, thereby reducing unnecessary physical exertion.	moderate
Administrative	Work schedules were reorganized to limit continuous sitting to a maximum of two hours, with regular breaks based on individual physical capacity. Targeted stretching routines for commonly affected muscle groups (e.g., neck, shoulders, back, knees, and feet) were incorporated. The 5S methodology was applied to workspace organization, and clear safety protocols were established.	moderately low
PPE	PPE was introduced to reduce localized vibration, particularly to the feet, during sewing tasks. Workers were encouraged to wear appropriate gear that supports ergonomic performance and minimizes physical risk.	low

## Data Availability

The data supporting the findings of this study are not publicly available due to ethical and privacy restrictions, as they contain information that could compromise the confidentiality of the study participants. Anonymized datasets may be made available from the corresponding author upon reasonable request and subject to approval by the relevant ethics committee.

## Abbreviations

The following abbreviations are used in this manuscript:

FGD	Focus Group Discussion
HEC	Hierarchy of Ergonomic Controls
MSD	Musculoskeletal Disorder
MSU	Mahasarakham University
NASA-TLX	National Aeronautics and Space Administration Task Load Index
NMQ	Nordic Musculoskeletal Questionnaire
OWAS	Ovako Working Posture Analysis System
PHEP-SEA	Public Health and Environmental Policy in Southeast Asia
PPE	Personal Protective Equipment
REBA	Rapid Entire Body Assessment
RULA	Rapid Upper Limb Assessment
SPSS	Statistical Package for the Social Sciences
UBRU	Ubon Ratchathani Rajabhat University
WMSDs	Work-Related Musculoskeletal Disorders
WWL	Weighted Workload

## References

[1] Loewenson R. (2021). Rethinking the Paradigm and Practice of Occupational Health in a World Without Decent Work: A Perspective from East and Southern Africa. New Solut..

[2] Sverdlik A., Kothiwal K., Kadungure A., Agarwal S., Machemedze R., Verma S., Loewenson R. (2024). Understanding the interplay of occupational, public health, and climate-related risks for informal workers: A new framework with findings from Zimbabwe and India. Soc. Sci. Med..

[3] Gangopadhyay S., Das S., Banerjee S., Das S. (2022). Occupational Ergonomics and Industrial Hygiene for Evaluation of Health-Related Hazards in Informal Sectors and SMEs. Saf. Health Work..

[4] Su J.-M., Chang J.-H., Indrayani N.L.D., Wang C.-J. (2023). Machine learning approach to determine the decision rules in ergonomic assessment of working posture in sewing machine operators. J. Saf. Res..

[5] Okareh O.T., Solomon O.E., Olawoyin R. (2021). Prevalence of ergonomic hazards and persistent work-related musculoskeletal pain among textile sewing machine operators. Saf. Sci..

[6] Kanniappan V., Palani V. (2020). Prevalence of musculoskeletal disorders among sewing machine workers in a leather industry. J. Lifestyle Med..

[7] Das S., Krishna Moorthy M., Shanmugaraja K. (2023). Analysis of musculoskeletal disorder risk in cotton garment industry workers. J. Nat. Fibers.

[8] Tondre S., Deshmukh T. (2019). Guidelines to sewing machine workstation design for improving working posture of sewing operator. Int. J. Ind. Ergon..

[9] Hulshof C.T.J., Colosio C., Daams J.G., Ivanov I.D., Prakash K.C., Kuijer P.P.F.M., Leppink N., Mandic-Rajcevic S., Masci F., van der Molen H.F. (2019). WHO/ILO work-related burden of disease and injury: Protocol for systematic reviews of exposure to occupational ergonomic risk factors and of the effect of exposure to occupational ergonomic risk factors on osteoarthritis of hip or knee and selected other musculoskeletal diseases. Environ. Int..

[10] Chokprasit P., Yimthiang S., Veerasakul S. (2022). Predictors of Low Back Pain Risk among Rubber Harvesters. Int. J. Environ. Res. Public Health.

[11] Pamungkas R.A., Ruga F.B.P., Kusumapradja R. (2022). Impact of Physical Workload and Mental Workload on Nurse Performance: A Path Analysis. Int. J. Nurs. Health Serv..

[12] Braarud P.Ø. (2020). An efficient screening technique for acceptable mental workload based on the NASA Task Load Index—Development and application to control room validation. Int. J. Ind. Ergon..

[13] Dönmez K., Demirel S., Özdemir M. (2020). Handling the pseudo pilot assignment problem in air traffic control training by using NASA TLX. J. Air Transp. Manag..

[14] Basumerda C., Artsitella C.R., Setiawan D. (2023). Mental workload analysis of workers in the textile manufacturing company during the Covid-19 pandemic using NASA-TLX. Proceedings of the AIP Conference Proceedings.

[15] Galy E., Paxion J., Berthelon C. (2018). Measuring mental workload with the NASA-TLX needs to examine each dimension rather than relying on the global score: An example with driving. Ergonomics.

[16] Lyon B.K., Popov G. (2019). Risk treatment strategies: Harmonizing the hierarchy of controls and inherently safer design concepts. Prof. Saf..

[17] Ajslev J.Z.N., Møller J.L., Andersen M.F., Pirzadeh P., Lingard H. (2022). The Hierarchy of Controls as an Approach to Visualize the Impact of Occupational Safety and Health Coordination. Int. J. Environ. Res. Public Health.

[18] Hoonakker P., Carayon P., Gurses A., Brown R., McGuire K., Khunlertkit A., Walker J.M. (2011). Measuring workload of ICU nurses with a questionnaire survey: The NASA Task Load Index (TLX). IIE Trans. Healthc. Syst. Eng..

[19] Yavuz Ş., Gür B., Çakır A.D., Köse D.A. (2021). investigation of the posture positions of the apparel workshop employees with the REBA and RULA method. Hittite J. Sci. Eng..

[20] Nugraha A., Widajati N., Guan N.Y., Febriyanto E.C., Oktavia N.D. (2024). Risk Analysis of Work Posture and Body Mass Index to Musculoskeletal Disorders among Librarians at Universitas Airlangga. Indones. J. Occup. Saf. Health.

[21] Nayak G.K., Kim E. (2021). Development of a fully automated RULA assessment system based on computer vision. Int. J. Ind. Ergon..

[22] Akyeampong J., Udoka S., Caruso G., Bordegoni M. (2014). Evaluation of hydraulic excavator Human–Machine Interface concepts using NASA TLX. Int. J. Ind. Ergon..

